# Aerobic Physical Activity and Depression Among Patients With Cancer: A Systematic Review and Meta-Analysis

**DOI:** 10.1001/jamanetworkopen.2024.37964

**Published:** 2024-10-08

**Authors:** Matthew Kulchycki, Henry Ratul Halder, Nicole Askin, Rasheda Rabbani, Fiona Schulte, Maya M. Jeyaraman, Lillian Sung, Deepak Louis, Lisa Lix, Allan Garland, Alyson L. Mahar, Ahmed Abou-Setta, Sapna Oberoi

**Affiliations:** 1 Rady Faculty of Health Sciences, University of Manitoba, Winnipeg, Manitoba, Canada; 2 Department of Community Health Sciences, University of Manitoba, Winnipeg, Manitoba, Canada; 3 Surveillance and Reporting, Cancer Advanced Analytics, Cancer Research and Analytics, Cancer Care Alberta, Alberta Health Services, Calgary, Alberta, Canada; 4 Neil John Maclean Health Sciences Library, University of Manitoba, Winnipeg, Manitoba, Canada; 5 George & Fay Yee Center for Healthcare Innovation, University of Manitoba, Winnipeg, Manitoba, Canada; 6 Division of Psychosocial Oncology, Department of Oncology, Cumming School of Medicine, University of Calgary, Calgary, Alberta, Canada; 7 Division of Haematology/Oncology, The Hospital for Sick Children, Toronto, Ontario, Canada; 8 Department of Pediatrics and Child Health, University of Manitoba, Winnipeg, Manitoba, Canada; 9 Department of Internal Medicine, Rady Faculty of Health Sciences, University of Manitoba, Winnipeg, Manitoba, Canada; 10 School of Nursing, Queen’s University, Kingston, Ontario, Canada; 11 Division of Cancer Care and Epidemiology, Queen’s Cancer Research Institute, Queen’s University, Kingston, Ontario, Canada; 12 Department of Pediatric Hematology-Oncology, CancerCare Manitoba, Winnipeg, Manitoba, Canada

## Abstract

**Importance:**

Depression is prevalent among patients with cancer, affecting their quality of life and survival. Aerobic physical activity (APA) is an effective strategy for managing depression in the general population, but its effectiveness for reducing depressive symptoms among patients with cancer requires further study.

**Objective:**

To evaluate whether APA decreases depression severity in patients with cancer by synthesizing data from published randomized clinical trials (RCTs).

**Data Sources:**

Six databases (MEDLINE, Embase, Cochrane Central Register of Controlled Trials, CINAHL, PsycINFO, and Scopus) were systematically searched for relevant citations published between January 1, 1980, and July 5, 2023.

**Study Selection:**

This systematic review and meta-analysis included RCTs comparing APA interventions with usual care, waitlist control, or attention control for managing depression in patients with cancer, irrespective of age and cancer type.

**Data Extraction and Synthesis:**

Two reviewers independently conducted screening and data extraction. Risk of bias was evaluated using the Cochrane Risk of Bias Tool, version 2. The Preferred Reporting Items for Systematic Reviews and Meta-Analyses (PRISMA) reporting guideline was followed. Standardized mean differences (SMDs) were calculated using a random-effects model. Data collection and analyses were performed between June 2022 and March 2024.

**Main Outcomes and Measures:**

The primary outcome was severity of self-reported depression reported within 1 month of the end of intervention (short term). Secondary outcomes were severity of self-reported depression between 1 and 6 months post intervention (medium term) and between 6 and 12 months post intervention (long term).

**Results:**

This meta-analysis included 25 RCTs with a total of 1931 adults with cancer (age range, 18-80 years). Ten RCTs (40%) had low risk of bias. Aerobic physical activity was associated with decreased self-reported depression among adults with cancer across the 25 included trials (n = 1931 participants; SMD, −0.38 [95% CI, −0.59 to −0.18]; *P* < .001; *I*^2^ = 76%). This decrease in depression scores was also significant for the secondary outcomes of long-term depression across 3 trials (n = 299 participants; SMD, −0.32 [95% CI, −0.60 to −0.04]; *P* = .03; *I*^2^ = 31%) but not for medium-term depression across 2 trials (n = 143 participants; SMD, −0.27 [95% CI, −0.60 to 0.06]; *P* = .10; *I*^2^ = 0%).

**Conclusions and Relevance:**

In this systematic review and meta-analysis, APA was associated with modest short-term and long-term reductions of depression among adults with cancer. Future studies should discern the effectiveness of APA in combination with other strategies for managing depression across various populations of patients with cancer.

## Key Points

**Question:**

Is aerobic physical activity associated with a reduction of depression among patients with cancer?

**Findings:**

In this systematic review and meta-analysis of 25 randomized clinical trials with a total of 1931 adults with cancer, aerobic physical activity was associated with decreased depressive symptoms within 1 month after the intervention and between 6 and 12 months after the intervention compared with usual care, waitlist control, attention control, or no intervention.

**Meaning:**

These findings suggest that aerobic physical activity should be recommended to adults with cancer for the reduction of depression.

## Teaser

This systematic review and meta-analysis examines the association between aerobic physical activity and depression severity among patients with cancer.

## Introduction

A cancer diagnosis increases the risk of anxiety, depression, and emotional distress among patients. Depression prevalence among patients with cancer ranges from 13% to 27%, which is 2 to 3 times higher than the general population.^1^ Depression is associated with adverse outcomes in this context, including prolonged hospital stays, increased physical and emotional distress, heightened risk of suicide,^2^ poor adherence to treatment,^3^ increased mortality,^4,5^ and impaired quality of life.^6^ The etiology of depression is multifactorial.^7^ A cancer diagnosis may lead to psychosocial distress, demoralization, and anxiety and can evoke adjustment and stress-related disorders.^8^ Cancer treatments can cause distressing adverse effects such as hair loss, weight changes, functional limitations, infertility and sexual dysfunction, or biochemical imbalances, predisposing patients to depressive episodes.^7^

The American Society of Clinical Oncology recommends psychological interventions such as cognitive behavioral therapy, structured physical activity, and antidepressants for managing depression in patients with cancer.^1^ The inclusion of structured physical activity in these guidelines relies on findings from 4 systematic reviews.^9,10,11,12^ Notably, only 1 of these reviews, focusing on patients with breast cancer, illustrated a reduction of depressive symptoms with physical activity, underscoring the scarcity of robust evidence supporting these recommendations.^11^ A recent meta-analysis indicates that exercise leads to moderate improvements in depressive symptoms in the general population, especially in individuals meeting clinical criteria for major depression, with more vigorous activities such as jogging showing larger effect sizes.^13^ In the context of patients with cancer, only 2 reviews have specifically examined aerobic physical activity (APA) interventions, with one focusing solely on patients with certain hematologic malignant neoplasms and another on breast cancer survivors.^3,9^ Given the accessibility of APA and its low requirements for specific supervision, equipment, or structure, there is growing interest in exploring the association between APA and depression across all cancer types. Furthermore, 150 minutes per week of moderate to vigorous APA is recommended for cardiovascular benefits, which may also have implications for managing depression in patients with cancer.^14^ Since the publication of previous reviews, additional randomized clinical trials (RCTs) investigating the role of APA in reducing depression among patients with cancer have been conducted.^15,16,17,18,19,20,21^ In addition to the aforementioned limitations, the previous reviews did not thoroughly explore potential factors contributing to differences in intervention effectiveness across trials.^9,22,23^

In this systematic review and meta-analysis, we aimed to assess the evidence regarding the association between APA and self-reported depression severity among patients with a cancer diagnosis to inform clinical practice in this context. Our primary objective was to ascertain whether engaging in APA is associated with a reduction of depression among patients with cancer. The secondary objective was to analyze patient, cancer, intervention, and methodology-related factors in the association between APA and depression severity in this population.

## Methods

This systematic review and meta-analysis was registered with PROSPERO (CRD42022333805). Because the data used for the systematic review were sourced from publicly available datasets, institutional human research ethics board approval was not required in accordance with the Common Rule. The study adhered to the *Cochrane Handbook for Systematic Reviews of Interventions* guidelines^24^ and followed the Preferred Reporting Items for Systematic Reviews and Meta-Analyses (PRISMA) reporting guideline.

### Search Strategy and Study Selection

We searched the MEDLINE, Embase, Cochrane Central Register of Controlled Trials, CINAHL, PsycINFO, and Scopus databases for eligible trials published between January 1, 1980, and July 5, 2023. The search strategy was developed by a librarian (N.A.) with expertise in searching databases for systematic reviews and included a combination of MeSH (Medical Subject Heading) terms and text words to identify relevant RCTs, focusing on physical activity interventions to reduce depression among patients with cancer (eTable 1 in Supplement 1). We reviewed the reference lists of existing systematic reviews to identify any additional eligible trials. Studies were included if they were RCTs that compared the effect between APA (regardless of type, intensity, or frequency) and standard or usual care, no intervention, waitlist control, or attention control on depression among patients of all ages, cancer types, and stages, including both those undergoing cancer treatment and cancer survivors. Data from eligible crossover RCTs were included prior to the crossover phase. We excluded abstract-only publications, quasi-randomized studies, cluster-randomized trials, studies with less than 80% of participants diagnosed with cancer, and non–English-language studies.

Two independent reviewers (M.K. and H.R.H.), blinded to each other, evaluated titles and abstracts in duplicate to identify potentially relevant publications. Any citation deemed potentially relevant by either reviewer underwent full-text retrieval and assessment for eligibility. The final inclusion of trials was based on consensus between the 2 reviewers, with any disagreements resolved through discussion and consultation with a third reviewer (S.O.) when necessary.

### Outcomes

The primary outcome was the severity of self-reported depression measured by self-report depression scales reported within 1 month post intervention (short term). When studies reported multiple measurements within this time frame, we extracted the measurement closest to the end of intervention for analysis. Secondary outcomes included the severity of self-reported depression assessed between 1 and 6 months post intervention (medium term) and between 6 and 12 months post intervention (long term). For studies with multiple assessments within these time frames, we prioritized the measurement closest to the end of the intervention. To ensure consistency across studies, we a priori defined a hierarchy of self-reported depression scales to determine which scale to prioritize for data collection and analysis from studies that measured depression using more than 1 scale (eTable 2 in Supplement 1).

### Intervention, Control Groups, and Definitions

For this review, APA intervention encompassed any cardiorespiratory or aerobic exercise (eg, cycling, running, jogging, walking, stationary bike or elliptical use, or aerobics), characterized by regular, rhythmic, continuous, and purposeful exercise involving the major muscle groups, with no restrictions on intensity, frequency, or duration.^25^ The mode of intervention delivery was categorized as supervised if conducted in the presence of an instructor or coach, whether in person or remotely. Group delivery involved simultaneous participation of multiple individuals. Weekly APA minutes were divided at 150 minutes, aligning with recommendations for low- to moderate-intensity exercise in healthy adults.^25^ Intensity of APA was defined based on metabolic equivalents (METs) using the American College of Sports Medicine approach: low (<3.0 METs), moderate (3.0-5.9 METs), and high (≥6.0 METs). If the study reported METs, this was used to classify the intensity of APA. If METs were not reported in a given study, intensity was determined using the 2011 Compendium of Physical Activities.^26^ In the studies using mixed aerobic interventions (eg, combined walking and swimming) or participants choosing different aerobic activities in the same trial, the intensity was listed as “not reportable.” We categorized the control group type as usual care, waitlist control, or attention control, in which (1) waitlist control participants received the intervention after outcomes were measured and (2) attention control participants reduced the confounding effects of interpersonal interactions by providing similar levels of socialization that the intervention group may have incidentally received.^27,28^

### Data Extraction and Risk-of-Bias Assessment

Two reviewers (M.K. and H.R.H.) independently extracted data from included trials using structured data extraction forms. Study-level variables included publication and enrollment years, study country, participant ages (adults vs children), sex (female vs male), cancer type, cancer stage (nonmetastatic, metastatic, or both), timing of intervention (during cancer treatment, after treatment, or both), inclusion of depression as an eligibility criterion for trial using study-defined thresholds (yes vs no), intervention details (eg, type of aerobic intervention), intervention mode (supervised, nonsupervised, or both), intervention format (group vs nongroup), intervention setting (in person, virtual, or both), weekly intervention duration (≥150 vs <150 minutes), intervention intensity in METs (low, moderate, or high), details of the comparator group, and outcome measures (including depression scales, and depression scores up to 1 month, between 1 and 6 months, and between 6 months and 1 year post intervention). Authors of studies with missing outcome data were contacted by email to provide relevant outcome data. Data from multiple publications of the same trial were consolidated.

Two reviewers (M.K. and H.R.H.) independently appraised the quality of included RCTs using the Cochrane Risk of Bias Tool, version 2.^29^ Based on this tool, each RCT was categorized as having low risk of bias, some concerns, or high risk of bias based on 5 domains: randomization process, deviations in the intended interventions, missing outcome data, outcome measurement, and selection of the reported results.^29^

### Statistical Analysis

Data from individual studies were pooled at the study level. The primary objective was to compare APA vs control (usual care, waitlist, or attention control) using various depression scales within 1 month post intervention. Data were synthesized using standardized mean differences (SMDs), in which SMDs less than 0 indicated mean depressive scores that were lower (better outcomes) in the APA group compared with the control group. Effects were weighted by the inverse variance using a random-effects model due to studies using various scales measuring depression.^30^ Pooled analyses by the 3 most frequent depression scales used by the studies were calculated using weighted mean differences (WMDs). Statistical heterogeneity between trials was assessed using the *I*^2^ statistic, which indicated the percentage of total variation across studies attributed to heterogeneity rather than chance.^30^ Statistical significance was determined as 2-tailed *P* < .05. Subgroup analyses for the primary outcome were conducted to examine potential variations in the effect size for APA based by cancer type, cancer stage, intervention timing, supervision, delivery mode, setting, intervention duration, weekly APA minutes, intensity, depression scale, and risk of bias. Additionally, we performed secondary analyses using methods similar to those for the primary outcome for depression scores reported between 1 and 6 months post intervention and between 6 months and 1 year post intervention. Meta-regression was used to assess the association between overall intervention duration and weekly minutes of intervention on outcomes. Potential publication bias was explored with visual inspection of funnel plots and with the Egger test for asymmetry. Meta-analyses were conducted using Review Manager, version 5.4 (Cochrane Collaboration),^31^ and R, version 4.3.2 (R Project for Statistical Computing), with the general meta and meta packages.^32^ Data collection and analyses were performed from June 2022 to March 2024.

## Results

### Characteristics of Included Studies

**Figure 1.**
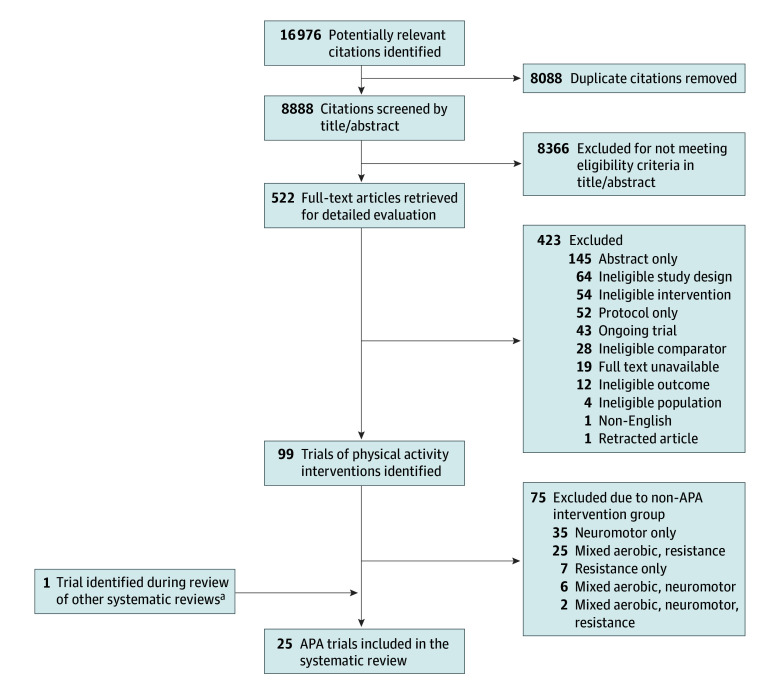
Flow Diagram of Trial Identification and Selection APA indicates aerobic physical activity. ^a^This study had depression indexed as a “psychological function,” which was not captured by the search strategy.^33^

Our search strategy identified 8888 unique citations; of these, 522 full-text articles were assessed for eligibility (Figure 1). A total of 24 trials^15,16,17,18,19,20,21,34,35,36,37,38,39,40,41,42,43,44,45,46,47,48,49,50^ met our inclusion criteria. Additionally, 1 eligible trial^33^ was identified from the reference lists of previous reviews^15,17^ and was included, bringing the total to 25 trials; our search strategy did not initially capture this trial because it indexed depression as a psychological function.

The 25 trials encompassed a total of 1931 adult patients (age range, 18-80 years; Figure 1), with 1011 (52%) assigned to the intervention group and 920 (48%) to the control group. Fourteen studies (56%) enrolled exclusively female patients,^18,21,34,35,37,38,39,40,41,43,45,46,47,50^ whereas only 1 study (4%) exclusively included male patients.^19^ In the 10 remaining trials,^15,16,17,20,33,36,42,44,48,49^ female patients constituted 47% of the study population (Table 1). Breast cancer (12 trials [48%]) was the most frequent diagnosis,^21,34,35,37,38,39,41,43,45,46,47,50^ followed by lung cancer (2 trials [8%]),^15,36^ prostate cancer (2 trials [8%]),^19,48^ and mixed cancer types (2 trials [8%]).^33,40^ Nine studies (36%) included patients not undergoing cancer treatment,^20,21,33,34,37,43,45,47,50^ 7 (28%) included patients undergoing treatment,^15,16,17,19,38,44,48^ and 9 (36%) included both (Table 1).^18,35,36,39,40,41,42,46,49^ No trials exclusively included patients receiving palliative care. Trials were conducted across 10 countries, with the US being the most prevalent (9 trials [40%]).^18,21,33,40,41,43,45,48,50^ A total of 12 trials (48%) were published after 2012.^15,16,17,18,19,20,21,34,35,36,37,38^

**Table 1.** Characteristics of the 25 Randomized Clinical Trials Included in the Systematic Review

Study	Age range, y	Country	Cancer diagnosis	Cancer stage	Phase of cancer treatment	Total No. of patients	Sex, %		Intervention							
							Female	Male	Type	Delivery	Setting	Supervised	No. of wk	Time per wk, min	Control group type
Rehman et al,^15^ 2023	20-55	Pakistan	Lung	Nonmetastatic	Undergoing treatment	40	40	60	Cycle ergometer	NR	In person	Yes	4	150	Usual care
Eisenhut et al,^16^ 2022	18-75	Switzerland	Brain	Nonmetastatic	Undergoing treatment	29	NR	NR	Treadmill or bicycle	Individual and group	In person	Yes	6	70-90	Attention
Piraux et al,^17^ 2022	>18	Belgium	Rectal	Nonmetastatic	Undergoing treatment	18	28	72	Cycle ergometer	Individual	In person	Yes	5	78-120	Usual care
Cartmel et al,^18^ 2021	18-75	US	Ovarian	Nonmetastatic and metastatic	Both^a^	144	100	0	APA	Individual	In person	No	24	150	Attention
Piraux et al,^19^ 2021	NR	Belgium	Prostate	Nonmetastatic	Undergoing treatment	78	0	100	HIIT via cycle ergometer	Individual	In person	Yes	5-8	24-45	Usual care
Adams et al,^20^ 2018	18-70	Canada	Testicular	Nonmetastatic	Not undergoing treatment	63	NR	NR	HIIT via uphill treadmill walking or running	Individual	In person	Yes	12	105	Usual care
Carter et al,^21^ 2018	27-74	US	Breast	Nonmetastatic	Not undergoing treatment	27	100	0	Walking on treadmill	NR	In person	Both^b^	12	150	Usual care
Gokal et al,^34^ 2016	NR	UK	Breast	Nonmetastatic	Not undergoing treatment	50	100	0	Walking	Individual	In person	No	12	50-150	Usual care
Ho et al,^35^ 2016	NR	China	Breast	Nonmetastatic	Both	147	100	0	Dance	Group	In person	Yes	3	180	Waitlist
Chen et al,^36^ 2015	NR	Taiwan	Lung	Nonmetastatic and metastatic	Both	116	53	47	Walking	Individual	In person	No	12	120	Usual care
Cantarero-Villaneuva et al,^37^ 2013	25-65	Spain	Breast	Nonmetastatic	Not undergoing treatment	68	100	0	Aquatic exercise	Group	In person	Yes	8	180	Usual care
Ergun et al,^38^ 2013	NR	Turkey	Breast	NR	Undergoing treatment	60	100	0	Walking	Individual	In person	No	12	90	Usual care
Saarto et al,^39^ 2012	35-68	Finland	Breast	Nonmetastatic	Both	573	100	0	Aerobics, circuit training, walking, Nordic walking	Individual	In person	Both^c^	52	180	Usual care
Dodd et al,^40^ 2010	NR	US	Mixed (>1 type)	Nonmetastatic and metastatic	Both	119	100	0	Walking, jogging, bicycling	Individual	In person	No	16-24	60-150	Waitlist
Cadmus et al,^41^ 2009	35-75	US	Breast	Nonmetastatic	Both	50	100	0	Walking and “other APA”	NR	In person	No	26	150	Usual care
Courneya et al,^42^ 2009	18-80	Canada	Lymphoma	Nonmetastatic and metastatic	Both	122	41	59	Cycle ergometer	NR	In person	Yes	12	45-135	Waitlist
Latka et al,^43^ 2009	34-79	US	Breast	Nonmetastatic	Not undergoing treatment	75	100	0	Walking and “other forms of APA”	NR	In person and virtual	Yes	26	150	Usual care
Chang et al,^44^ 2008	NR	Taiwan	Leukemia	Nonmetastatic	Undergoing treatment	12	45	55	Walking	NR	In person	Yes	3	60	Usual care
Payne et al,^45^ 2008	56-78	US	Breast	NR	Not undergoing treatment	20	100	0	Walking	NR	In person	No	14	80	Usual care
Courneya et al,^46^ 2007	25-78	Canada	Breast	Nonmetastatic	Both	102	100	0	Cycle ergometer, treadmill, elliptical	Individual	In person	Yes	20	45-135	Usual care
Daley et al,^47^ 2007	18-65	UK	Breast	Nonmetastatic	Not undergoing treatment	108	100	0	Walking, stepping, cycling, rowing	Individual	In person	Yes	8	150	Usual care
Monga et al,^48^ 2007	62-80	US	Prostate	Nonmetastatic	Undergoing treatment	21	NR	NR	Walking on treadmill	NR	In person	Yes	8	142.5	Usual care
Courneya et al,^49^ 2003	NR	Canada	Colorectal	Nonmetastatic and metastatic	Both	242	42	58	Walking, swimming, cycling	Individual	In person	No	16	60-150	Usual care
Pinto et al,^50^ 2003	NR	US	Breast	Nonmetastatic	Not undergoing treatment	24	100	0	Treadmill walking, arm or leg ergometer, stationary rowing	Individual	In person	Yes	12	150	Waitlist
Burnham and Wilcox,^33^ 2002	40-65	US	Mixed (>1 type)	Nonmetastatic	Not undergoing treatment	21	83	17	Treadmill, stationary cycling, stair-climbing machines	NR	In person	Yes	10	42-96	Usual care

Abbreviations: APA, aerobic physical activity; HIIT, high-intensity interval training; NR, not reported.

^a^ Includes participants both undergoing and not undergoing cancer treatment.

^b^ Supervised during the first 6 weeks, then tapered to home-based exercise in the following 6 weeks.

^c^ Weekly supervised sessions in addition to home-based exercise.

Of the 25 trials, 21 (84%) used self-reported depression instruments validated in oncology patients.^15,16,17,18,19,20,21,34,35,36,37,38,39,40,41,42,43,45,46,47,48,49^ The most common instrument used was the Center for Epidemiologic Studies Depression Scale (CES-D) (11 trials [44%]),^17,18,19,20,40,41,42,43,45,46,49^ followed by the Hospital Anxiety and Depression Scale (HADS) (5 trials [20%]),^15,21,34,35,36^ the Beck Depression Inventory (BDI) (4 trials [16%]),^16,38,39,48^ and the BDI Version II (BDI-II) (1 trial [4%]).^47^ The remaining 4 trials (16%) used nonvalidated scales,^33,37,44,50^ including the Profile of Mood States (POMS), the POMS Short Form (POMS-SF), and the Linear Analog Scale Assessment (LASA) (eTable 2 in Supplement 1).^51^ A depression diagnosis was not a prerequisite for enrollment in all studies. Most trials used mixed forms of APA intervention, whereas 7 (28%) focused on walking.^21,34,36,38,44,45,48^ Depression was the primary outcome in 9 studies (28%)^16,18,21,34,35,36,44,45,50^ and the secondary outcome in 16 studies (56%).^15,17,19,20,37,38,39,40,41,42,43,46,47,48,49,51^ Among the 25 RCTs, the comparator group comprised usual care in 19 studies (76%),^15,17,19,20,21,33,34,36,37,38,39,41,43,44,45,46,47,48,49^ waitlist control in 4 studies (16%),^35,40,42,50^ and attention control in 2 studies (8%).^16,18^ The attention control of 1 study^18^ comprised weekly phone calls to participants to discuss cancer health education. The attention control for another study^16^ consisted of participants meeting twice weekly in supervised groups to share daily experiences without performing physical activity or psychotherapy.

### Outcomes

#### Primary Outcome

**Figure 2.**
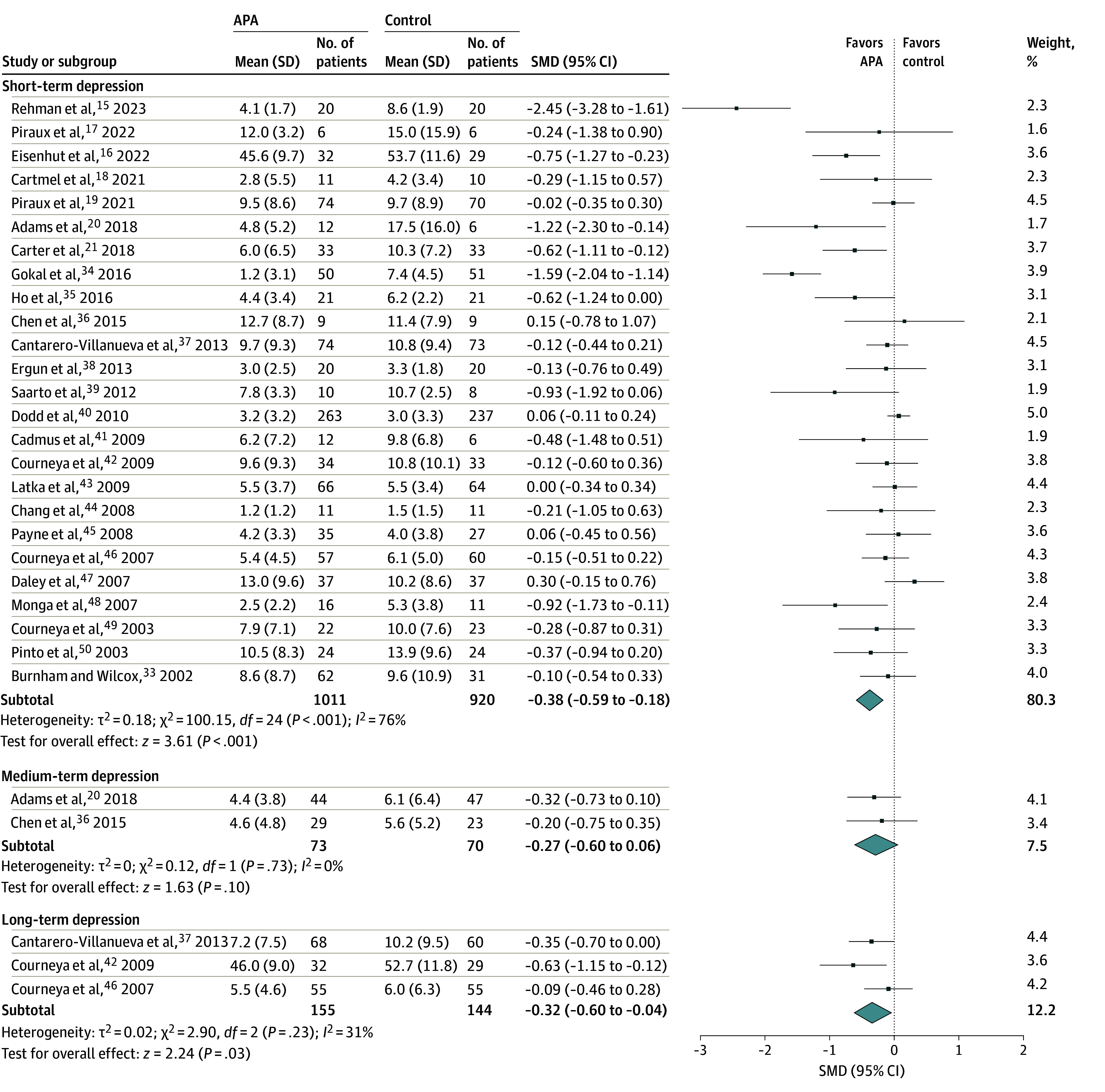
Forest Plot of the Association of Aerobic Physical Activity (APA) With Short-Term, Medium-Term, and Long-Term Depression Diamonds refer to the aggregate standardized mean difference (SMD) and 95% CI for that subgroup. Square size represents the relative weight of the study. Subtotals refer to overall effect in SMD with 95% CI.

The pooled results from 25 trials indicated that APA interventions were associated with a statistically significant decrease in short-term depression scores within 1 month post intervention despite substantial heterogeneity (n = 1931 participants; SMD, −0.38 [95% CI, −0.59 to −0.18]; *P* < .001; *I*^2^ = 76%) (Figure 2). Stratified analysis of short-term depression scores by control type did not reveal any statistically significant differences for the usual care and waitlist subgroups (n = 23 trials; SMD, −0.39 [95% CI, −0.62 to −0.17]; *P* < .001; *I*^2^ = 77%) or attention control subgroups (n = 2 trials; SMD, –0.35 [95% CI, −1.20 to 0.50]; *P* = .42; *I*^2^ = 65%) (*P* for subgroup difference = .92). To address potential measurement bias from nonvalidated self-depression scales (eg, POMS, POMS-SF, and LASA) used in 4 trials,^33,37,44,50^ a sensitivity analysis was conducted by excluding these trials. An SMD of −0.35 (95% CI, −0.57 to −0.12; *P* = .002; *I*^2^ = 78%) was observed for the estimated effect size of the association between APA intervention and depression among the remaining 21 studies^15,16,17,18,19,20,21,34,35,36,37,38,39,40,41,42,43,45,46,47,48,49^ using validated scales (eFigure 4 in Supplement 1). The pooled analyses by the 3 most frequently validated depression scales used by the studies indicated a statistically significant reduction in short-term depressive symptoms associated with APA compared with the control when the HADS was used (n = 5 studies; WMD, −3.07 [95% CI, −5.42 to −0.71]; *P* = .01; *I*^2^ = 92%) and not when the CES-D (n = 11 studies; WMD, −0.44 [95% CI, −1.37 to 0.49]; *P* = .36; *I*^2^ = 0%) or the BDI (n = 4 studies; SMD, −0.46 [95% CI, −1.56 to 0.63]; *P* = .41; *I*^2^ = 47%) was used.

**Figure 3.**
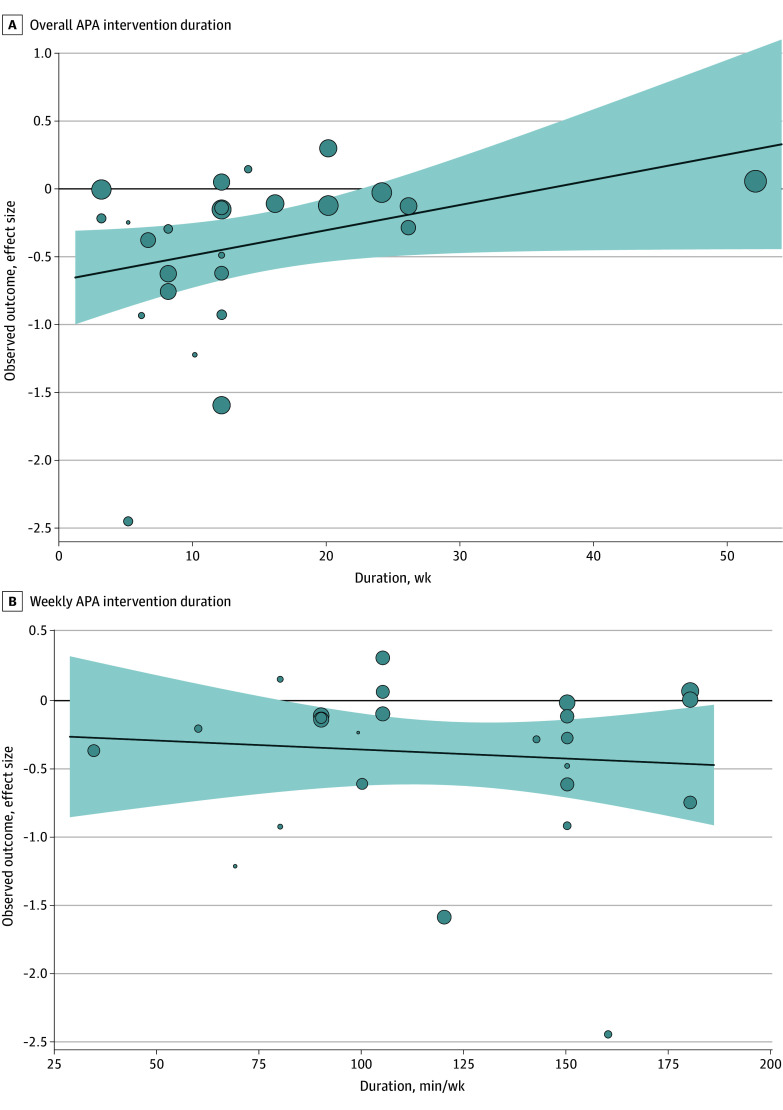
Meta-Regression Analyses of Aerobic Physical Activity (APA) Duration A, Association of APA intervention duration with effect size. The solid line represents linear estimates for the standardized mean difference (SMD) as a function of the weekly minutes of APA at the group level. The shaded area represents the 95% CIs. Bubbles are the observed SMD of each study, with bubble sizes proportional to the study weights. B, Association of weekly APA intervention duration with effect size. The solid line represents linear estimations for SMD as a function of the weekly minutes of APA at the group level. The shaded area represents the 95% CIs. Bubbles are the observed SMD of each study, with bubble sizes proportional to study weights.

Subgroup analyses revealed no statistically significant differences based on cancer type and treatment characteristics, including cancer diagnosis and stage and timing of cancer treatment (Table 1). No statistically significant differences were observed between studies for short-term depression outcomes in subgroup analyses based on intervention characteristics such as intervention supervision, delivery, mode, intensity, and weekly intervention minutes (Table 1). Meta-regression analysis of overall APA intervention duration did not reach statistical significance (β [SE], 0.02 [0.01]; *P* = .06) (Figure 3A). Additionally, the meta-regression of weekly intervention minutes showed no association with control heterogeneity (β [SE], −0.001 [0.003]; *P* = .66) (Figure 3B). A post hoc subgroup analysis examining differences in effect size among the 3 most commonly used depression assessment scales (CES-D, HADS, and BDI) revealed no statistically significant differences based on the type of scale used (Table 2 and eFigure 5 in Supplement 1).

**Table 2.** Association of APA Intervention With Short-Term Depression by Patient, Cancer, Intervention, and Methodology-Related Factors

Subgroup	No. (%) of studies (n = 25)^a^	No. (%) of participants (n = 1931)	SMD (95% CI)	*I*^2^, %	*P* value for subgroup difference
Cancer diagnosis					
Breast	12 (48)	1161 (60)	−0.25 (−0.44 to −0.06)	49	.70
Nonbreast	11 (44)	678 (35)	−0.41 (−0.73 to −0.09)	72	
Mixed (>1 type)	2 (8)	92 (5)	−0.38 (−1.86 to 1.11)	85	
Cancer stage					
Nonmetastatic	20 (80)	1458 (76)	−0.37 (−0.59 to −0.15)	69	.81
Metastatic	0	0	NA	NA	
Both	4 (16)	455 (24)	−0.46 (−1.12 to 0.21)	92	
Cancer treatment phase					
Receiving cancer treatment	8 (32)	275 (14)	−0.50 (−1.07 to 0.07)	79	.08
Not receiving cancer treatment	9 (36)	379 (20)	−0.45 (−0.72 to −0.18)	36	
Both	8 (32)	1277 (66)	−0.11 (−0.26 to 0.04)	36	
Intervention supervision					
Supervised	15 (60)	847 (44)	−0.43 (−0.69 to −0.17)	66	.36
Nonsupervised	8 (32)	557 (29)	−0.18 (−0.42 to 0.07)	46	
Both	2 (8)	527 (27)	−0.35 (−1.30 to 0.60)	81	
Intervention delivery					
Group	3 (12)	205 (11)	−0.44 (−1.06 to 0.19)	70	.42
Individual	14 (56)	1313 (68)	−0.31 (−0.56 to −0.06)	45	
Intervention mode					
In person	16 (64)	1307 (68)	−0.44 (−0.70 to −0.19)	73	.25
Virtual	0	0	NA	NA	
Both	1 (4)	67 (3)	−0.12 (−0.60 to 0.36)	NA	
Intervention duration, wk					
<12	10 (40)	420 (22)	−0.66 (−1.09 to −0.23)	73	.02
≥12	15 (60)	1467 (76)	−0.13 (−0.28 to 0.02)	38	
Weekly intervention duration, min/wk					
<150	15 (60)	833 (43)	−0.22 (−0.39 to −0.04)	27	.20
≥150	10 (40)	1098 (57)	−0.46 (−0.78 to −0.13)	81	
Intensity, METs					
Low, <3.0	0	0	NA	NA	.68
Moderate, 3.0-5.9	6 (24)	250 (13)	−0.46 (−0.74 to −0.19)	10	
High, ≥6.0	4 (16)	573 (30)	−0.71 (−1.86 to 0.43)	91	
Validated depression scale used					
CES-D	11 (44)	827 (43)	−0.07 (−0.21 to 0.07)	0	.07
HADS	5 (20)	340 (18)	−1.08 (−1.94 to −0.22)	92	
BDI	4 (16)	579 (30)	−0.12 (−0.45 to 0.22)	33	
Overall risk of bias					
Low	10 (40)	1152 (60)	−0.28 (−0.56 to 0.00)	76	.76
Some concerns	8 (32)	459 (24)	−0.30 (−0.52 to −0.07)	25	
High	7 (28)	320 (17)	−0.53 (−1.15 to 0.08)	84	

Abbreviations: APA, aerobic physical activity; BDI, Beck Depression Inventory; CES-D, Center for Epidemiologic Studies Depression Scale; HADS, Hospital Anxiety and Depression Scale; MET, metabolic equivalent; NA, not applicable; SMD, standardized mean difference.

^a^ Values may not sum to 100% due to missing data.

On the risk-of-bias assessment, 10 studies (40%) had a low overall risk of bias, 8 (32%) had some concerns about overall bias, and 7 (28%) had a high overall risk (eFigures 1 and 2 in Supplement 1). For risk of bias in the randomization process, 15 studies (60%) had low risk, 9 (36%) showed some concerns, and 1 (4%) had high risk. Risk of bias associated with deviations in intended interventions was low for 21 studies (84%). All trials but 1 (24 [96%]) had low risk of bias or some concerns for risk of bias related to missing outcome data. A subgroup analysis by overall risk of bias (low, some concerns, high) did not yield a statistically significant difference in effect size (Table 2). Visual inspection of the funnel plot for publication bias did not reveal apparent asymmetry (eFigure 3 in Supplement 1); however, the Egger test indicated a significant presence of publication bias (bias estimate [SE], −2.2 [0.78]; *t* value, −2.8; *df* = 23; *P* = .01).^52^

#### Secondary Outcomes

When data from 2 studies were pooled, APA intervention was not associated with statistically significantly lower depressive symptom scores than the control group between 1 and 6 months post intervention (n = 143 participants; SMD, −0.27 [95% CI, −0.60 to 0.06]; *P* = .10; *I*^2^ = 0%). Nevertheless, when data were pooled from 3 studies, there was a statistically significant decrease in depression scores between 6 months and 1 year post intervention (n = 299 participants; SMD, −0.32 [95% CI, −0.60 to −0.04]; *P* = .03; *I*^2^ = 31%) with limited heterogeneity (Figure 2).

## Discussion

In this meta-analysis of 25 RCTs involving 1931 adults with cancer, APA interventions were associated with a statistically significant decrease in both short-term and long-term depression. The SMD of −0.38 indicates a small effect size.^53,54^ This effect size was consistent across the short-term, medium-term, and long-term assessments, bolstering our confidence in the efficacy of the intervention. When studies using HADS scores were examined independently, the association with a decrease in short-term depressive scores remained statistically significant. Given that the minimum clinically important difference (MCID) for the HADS among the cancer population has not been explicitly defined in the existing literature, we compared our findings to the established MCID range of 1.4 to 1.7 for the HADS in patients with chronic obstructive pulmonary disease and cardiovascular disease.^55,56^ The observed decrease of 3.1 points in short-term depression as measured by the HADS exceeds the reported MCID, indicating a clinically meaningful effect size for the association of APA with depression. The observed positive association of APA with reduced depressive symptoms aligns with the findings of a recently published systematic review and meta-analysis, which identified aerobic activity as an effective intervention for adults with major depression in the general population.^57^ A slight reduction of depressive symptoms with APA was also shown in a previous systematic review of patients with hematologic malignant neoplasms, and a substantial decrease in depression with APA was demonstrated in subgroup analysis in a systematic review of patients with breast cancer.^3,9^ Given the additional benefit of APA for cancer-related fatigue, cognitive function, and health-related quality of life among adults with cancer, our findings suggest that APA should be recommended to adults with cancer who are experiencing depression, taking into account their ability to tolerate APA.^58,59^

Subgroup analyses indicated that the effect sizes observed for the association of APA interventions with short-term depression did not vary by type and stage of cancer and phase of cancer treatment, suggesting that aerobic activity may potentially be recommended for adults with cancer regardless of these factors. Similarly, subgroup analyses based on intervention characteristics did not show statistically significant differences by intervention format, setting, or intensity of the aerobic activity. The comparable effect size, irrespective of mode or supervision of intervention, alludes to the fact that unsupervised or individual aerobic interventions are also effective for depression. Because the subgroup analyses were exploratory, given the heterogeneity in reported intensity and the absence of trials using low-intensity APA interventions, these findings warrant attention in future studies. Meta-regression analysis of the overall duration of APA (measured in weeks) revealed no statistically significant effect size, indicating that benefits from APA may be achievable even with shorter intervention periods. Some studies appeared as clear outliers and may contribute to the effect size observed. Moreover, meta-regression analysis of the duration of APA per week revealed no statistically significant effect size, potentially implying that any level of engagement in APA, irrespective of the number of minutes, holds importance. Consistency in engaging in APA over time may potentially act as a positive mediator in reducing depressive symptoms and should be further investigated in future studies.

### Strengths and Limitations

This review has several strengths. Foremost, we adhered to a rigorous methodology, strictly following established guidelines for conducting systematic reviews. This adherence bolsters the reliability and credibility of our findings and ensures a robust framework for data synthesis and interpretation. Additionally, our comprehensive inclusion criteria, encompassing a diverse population of patients with cancer, helped identify essential gaps in the existing literature. The concern for publication bias remained low due to the absence of apparent asymmetry in the funnel plot and the potential for false-positive results with the Egger test, as indicated by previous reports.^60^

Our review has several limitations that warrant consideration. The observed unexplained heterogeneity in results can be attributed to the diverse range of APA interventions used. It was not feasible to explore the specific effects of individual aerobic activities on depression due to the limited number of studies for each subtype. The multifaceted etiology of depression, influenced by patient characteristics such as previous, current, or family history of depression or anxiety diagnosis, as well as cancer-related factors, including treatment types and associated toxicities, may affect the efficacy of aerobic activity for depression; the included trials did not comprehensively account for these factors. Also, the trials failed to specify a clinical diagnosis of depression, depressive symptom threshold, or antidepressant use at enrollment, indicating a need for further research to elucidate how these variables modify intervention effect estimates, resulting in heterogeneity. The generalizability of this review is limited for patients with metastatic cancers, as most trials only included patients with nonmetastatic cancers. Similarly, nearly half of the trials focused specifically on patients with breast cancer, thereby limiting the generalizability of the findings to individuals with other cancer diagnoses. The absence of trials including children with cancer highlights a research gap in pediatric oncology. Finally, the inclusion of only English-language articles has the potential to introduce bias and limit the generalizability of the findings, and the occurrence of adverse effects of APA was not recorded in this review.

## Conclusions

In this systematic review and meta-analysis, APA was associated with a small but statistically significant reduction of depressive symptoms among adults with cancer, providing more robust evidence to support the existing recommendation of promoting APA in this context. More research is warranted to ascertain the efficacy of APA compared with other established effective interventions for depression and in combination with other established effective interventions for reducing depression in patients with cancer. Future studies should also investigate how preexisting diagnoses of depression and more granular cancer treatment–related factors may modify the effects of APA on depressive symptoms. The findings of this review suggest that prioritizing trials among understudied populations, such as children with cancer and those with metastatic disease, is crucial for a more comprehensive understanding of the potential benefits of APA for depression in these populations.

## Notes

Supplement 1. **eTable 1.** Search Strategy **eTable 2.** Self-Reported Depression Assessment Scales Used in Included Trials **eFigure 1.** Risk-of-Bias Domains for Each Included Study as per Cochrane Risk-of-Bias Tool 2 **eFigure 2.** Summary of the Risk of Bias of Studies Included in the Systematic Review **eFigure 3.** Funnel Plot Assessing Publication Bias for Short-Term Reduction in the Severity of Depression **eFigure 4.** Forest Plot of Association of Aerobic Physical Activity (APA) With Short-Term Depression Including the Trials Using Validated Self-Reported Depression Scales **eFigure 5.** Forest Plot of Association of Aerobic Physical Activity (APA) With Short-Term Depression Comparing the 3 Most Frequently Validated Self-Reported Depression Scales Used by the Trials

Supplement 2.
Data Sharing Statement
