# Supplementary material for: Aerobic Physical Activity and Depression Among Patients With Cancer: A Systematic Review and Meta-Analysis
Source: JAMA Netw Open. 2024 Oct 8;7(10):e2437964. doi: 10.1001/jamanetworkopen.2024.37964 (PMC11581595; doi:10.1001/jamanetworkopen.2024.37964)
Supplement: Supplement 1. — eTable 1. Search Strategy eTable 2. Self-Reported Depression Assessment Scales Used in Included Trials eFigure 1. Risk-of-Bias Domains for Each Included Study as per Cochrane Risk-of-Bias Tool 2 eFigure 2. Summary of the Risk of Bias of Studies Included in the Systematic Review eFigure 3. Funnel Plot Assessing Publication Bias for Short-Term Reduction in the Severity of Depression eFigure 4. Forest Plot of Association of Aerobic Physical Activity (APA) With Short-Term Depression Including the Trials Using Validated Self-Reported Depression Scales eFigure 5. Forest Plot of Association of Aerobic Physical Activity (APA) With Short-Term Depression Comparing the 3 Most Frequently Validated Self-Reported Depression Scales Used by the Trials [file jamanetwopen-e2437964-s001.pdf]

Supplementary Online Content

Kulchycki M, Halder HR, Askin N, et al. Aerobic physical activity and depression among patients with cancer: a systematic review and meta-analysis. *JAMA Netw Open*. 2024;7(10):e2437964. doi:10.1001/jamanetworkopen.2024.37964

eTable 1. Search Strategy

eTable 2. Self-Reported Depression Assessment Scales Used in Included Trials

eFigure 1. Risk-of-Bias Domains for Each Included Study as per Cochrane Risk-of-Bias Tool 2

eFigure 2. Summary of the Risk of Bias of Studies Included in the Systematic Review

eFigure 3. Funnel Plot Assessing Publication Bias for Short-Term Reduction in the Severity of Depression

eFigure 4. Forest Plot of Association of Aerobic Physical Activity (APA) With Short-Term Depression Including the Trials Using Validated Self-Reported Depression Scales

eFigure 5. Forest Plot of Association of Aerobic Physical Activity (APA) With Short-Term Depression Comparing the 3 Most Frequently Validated Self-Reported Depression Scales Used by the Trials

This supplementary material has been provided by the authors to give readers additional information about their work.

© 2024 Kulchycki M et al. *JAMA Network Open.*

eTable 1. Search Strategy

Medline

		No. Results
1	exp neoplasms/ or cancer survivors/ or psycho-oncology/ or exp hematopoietic stem cell transplantation/ or stem cell transplantation/ or hematopoietic stem cell mobilization/	3739041
2	(cancer* or neoplas* or tumo?r* or malignan* or metasta* or oncogen* or oncolog* or psychooncolog* or sarcoma* or leuk?emi* or lymphoma* or hodgkin* or nonhodgkin* or carcino* or melanoma* or thymoma* or myeloma* or blastoma* or hepatoblastoma* or mesenchymoma* or mesothelioma* or hepatoma* or adenocarcinoma* or glioma*).ti,ab,kf	4317109
3	(HSCT or ((h?ematopoietic or h?emato-poietic) adj3 (transplant* or sct or bct or mobili#ation)) or pbsct or pbct or psct or ((peripheral or pbsc or pbscs) adj3 transplant*) or autohct or autopbsct or autopbsc or autopsc or cbsct or ((autologous or auto-logous or auto or allogeneic or allo-geneic or homologous or homo-logous) adj hct)).ti,ab,kf	44187
4	or/1-3	5046055
5	depression/ or exp depressive disorder/ or sadness/ or demoralization/ or mental health/ or happiness/ or hope/ or resilience, psychological/	302190
6	(depress* or dysthymi* or melanchol* or affective or mood or distress* or sadness or sad or hopeless* or demorali* or mental health or psych* health or wellbeing or wellness or contented or contentment or happiness or hope* or resilien*).ti,ab,kf	1049135
7	5 or 6	1096843
8	exp exercise/ or exp exercise movement techniques/ or exp exercise therapy/ or dancing/ or exp physical fitness/ or exp sports/ or aquatic therapy/	375589
9	(exercis* or fitness or physiotherap* or physio therap* or kinesiotherap* or aerobics or athletic* or workout or gym? or gymnasi* or work* out or (activity adj4 prescri*)).ti,ab,kf	462696
10	(physical* adj4 (activ* or fit* or condition* or recondition* or program* or train*)).ti,ab,kf	187835
11	(activ* adj4 (living or play* or gaming or game* or lifestyle or behavio?r* or transport* or commut* or transit* or travel* or aerobic*)).ti,ab,kf	177595
12	(crossfit* or HIIT or HIIE or plyometric* or personal train* or bodybuild* or body build* or weightlift* or (weight* adj2 lift*)).ti,ab,kf	8689
13	((train* or condition* or program* or session*) adj5 (weight* or strength* or enduranc* or resistance or athletic* or cardiovascular or aerobic* or interval* or flexibility or balance or muscle* or muscular or circuit or isometric* or isotonic* or agility or power or propriocept* or coordination or gait)).ti,ab,kf	158596

© 2024 Kulchycki M et al. *JAMA Network Open.*

14	(treadmill* or walk* or jog* or swim* or running or runner* or run or runs or marathon* or triathl* or dance or dancing or climb* or stair* or elliptical* or cycling or bicycl* or bike or bikes or calisthenic* or skateboard* or rowing or rower or canoe* or kayak* or paddl* or skate or skating or ski or skiing or hike or hiking or jump or jumping).ti,ab,kf	532856
15	(tai ji or tai chi or ai chi or yoga or pilates or stretch or stretches or stretching or flexibility or movement or qigong or qi gong or barre or tae bo or zumba or jazzercise).ti,ab,kf	435748
16	(exergam* or wii fit or zwift or peloton or kinect).ti,ab,kf	2516
17	(martial art* or boxing or kickboxing or karate or judo or jiu jitsu or jiujitsu or taekwondo or tae kwon do or fencing or wrestl* or aikido or fight* or grappl*).ti,ab,kf	48231
18	(sport* or golf* or aquatic* or gymnastic* or tennis or badminton or volleyball or ball or basketball or baseball or bowling or lacrosse or racquet* or ringette or softball or polo or cricket or hockey or football or soccer or rugby or handball or mountaineer*).ti,ab,kf	212520
19	or/8-18	1877837
20	randomized controlled trial/ or Random Allocation/ or Double Blind Method/ or Single Blind Method/	697529
21	("clinical trial, phase i" or "clinical trial, phase ii" or "clinical trial, phase iii" or "clinical trial, phase iv" or controlled clinical trial or multicenter study or clinical trial or pragmatic clinical trial).pt	856245
22	exp Clinical Trials as topic/	374140
23	(clinical adj trial$).tw,kf	447472
24	(RCT or RCTs).tw,kf	64729
25	((singl$ or doubl$ or treb$ or tripl$) adj (blind$3 or dumm* or mask$3)).tw,kf	188784
26	PLACEBOS/	35916
27	(placebo$ or sham).tw,kf	328095
28	(randomized or randomised or randomly).ab	990502
29	trial.ti	262853
30	or/20-29	2287124
31	exp animals/ not humans.sh	5010272
32	30 not 31	2070023
33	4 and 7 and 19 and 32	1944
	limit 33 to (english language and yr="1980 -Current")	1896

Embase

1	exp neoplasm/ or exp cancer patient/ or psycho-oncology/ or exp hematopoietic stem cell transplantation/ or stem cell transplantation/	5122112

© 2024 Kulchycki M et al. *JAMA Network Open.*

2	(cancer* or neoplas* or tumo?r* or malignan* or metasta* or oncogen* or oncolog* or psychooncolog* or sarcoma* or leuk?emi* or lymphoma* or hodgkin* or nonhodgkin* or carcino* or melanoma* or thymoma* or myeloma* or blastoma* or hepatoblastoma* or mesenchymoma* or mesothelioma* or hepatoma* or adenocarcinoma* or glioma*).ti,ab,kw	5586634
3	(HSCT or ((h?ematopoietic or h?emato-poietic) adj3 (transplant* or sct or bct or mobili#ation)) or pbsct or pbct or psct or ((peripheral or pbsc or pbscs) adj3 transplant*) or autohct or autopbsct or autopbsc or autopsc or cbsct or ((autologous or auto-logous or auto or allogeneic or allo-geneic or homologous or homo-logous) adj hct)).ti,ab,kw	82961
4	or/1-3	6458335
5	exp depression/ or sadness/ or demoralization/ or mental health/ or psychological well-being/ or happiness/ or hope/ or hopelessness/ or unhappiness/ or psychological resilience/	728081
6	(depress* or dysthymi* or melanchol* or affective or mood or distress* or sadness or sad or hopeless* or demorali* or mental health or psych* health or wellbeing or wellness or contented or contentment or happiness or hope* or resilien*).ti,ab,kw	1366632
7	5 or 6	1576315
8	exp exercise/ or training/ or exp physical activity/ or exp kinesiotherapy/ or dancing/ or fitness/ or exp sport/	1002836
9	(exercis* or fitness or physiotherap* or physio therap* or kinesiotherap* or aerobics or athletic* or workout or gym? or gymnasi* or work* out or (activity adj4 prescri*)).ti,ab,kw	603035
10	(physical* adj4 (activ* or fit* or condition* or recondition* or program* or train*)).ti,ab,kw	244515
11	(activ* adj4 (living or play* or gaming or game* or lifestyle or behavio?r* or transport* or commut* or transit* or travel* or aerobic*)).ti,ab,kw	219102
12	(crossfit* or HIIT or HIIE or plyometric* or personal train* or bodybuild* or body build* or weightlift* or (weight* adj2 lift*)).ti,ab,kw	10568
13	((train* or condition* or program* or session*) adj5 (weight* or strength* or enduranc* or resistance or athletic* or cardiovascular or aerobic* or interval* or flexibility or balance or muscle* or muscular or circuit or isometric* or isotonic* or agility or power or propriocept* or coordination or gait)).ti,ab,kw	201271
14	(treadmill* or walk* or jog* or swim* or running or runner* or run or runs or marathon* or triathl* or dance or dancing or climb* or stair* or elliptical* or cycling or bicycl* or bike or bikes or calisthenic* or skateboard* or rowing or rower or canoe* or kayak* or paddl* or skate or skating or ski or skiing or hike or hiking or jump or jumping).ti,ab,kw	681880
15	(tai ji or tai chi or ai chi or yoga or pilates or stretch or stretches or stretching or flexibility or movement or qigong or qi gong or barre or tae bo or zumba or jazzercise).ti,ab,kw	515095
16	(exergam* or wii fit or zwift or peloton or kinect).ti,ab,kw	3173

© 2024 Kulchycki M et al. *JAMA Network Open.*

17	(martial art* or boxing or kickboxing or karate or judo or jiu jitsu or jiujitsu or taekwondo or tae kwon do or fencing or wrestl* or aikido or fight* or grappl*).ti,ab,kw	57481
18	(sport* or golf* or aquatic* or gymnastic* or tennis or badminton or volleyball or ball or basketball or baseball or bowling or lacrosse or racquet* or ringette or softball or polo or cricket or hockey or football or soccer or rugby or handball or mountaineer*).ti,ab,kw	253244
19	or/8-18	2578851
20	exp randomized controlled trial/ or Randomization/ or single blind procedure/ or double blind procedure/ or triple blind procedure/	835470
21	exp "clinical trial (topic)"/	390735
22	(clinical adj trial$).tw,kw	623087
23	(RCT or RCTs).tw,kw	97030
24	((singl$ or doubl$ or treb$ or tripl$) adj (blind$3 or dumm* or mask$3)).tw,kw	263554
25	PLACEBO/	380253
26	(placebo$ or sham).tw,kw	470699
27	(randomized or randomised or randomly).ab	1391285
28	trial.ti	358290
29	or/20-28	2553590
30	(exp animal/ or nonhuman/) not exp human/	6815610
31	29 not 30	2247279
32	4 and 7 and 19 and 31	3897
33	limit 32 to (english language and yr="1980 -Current")	3825

Central

1	exp neoplasms/ or cancer survivors/ or psycho-oncology/ or exp hematopoietic stem cell transplantation/ or stem cell transplantation/ or hematopoietic stem cell mobilization/	89174
2	(cancer* or neoplas* or tumo?r* or malignan* or metasta* or oncogen* or oncolog* or psychooncolog* or sarcoma* or leuk?emi* or lymphoma* or hodgkin* or nonhodgkin* or carcino* or melanoma* or thymoma* or myeloma* or blastoma* or hepatoblastoma* or mesenchymoma* or mesothelioma* or hepatoma* or adenocarcinoma* or glioma*).ti,ab,kw	264556
3	(HSCT or ((h?ematopoietic or h?emato-poietic) adj3 (transplant* or sct or bct or mobili#ation)) or pbsct or pbct or psct or ((peripheral or pbsc or pbscs) adj3 transplant*) or autohct or autopbsct or autopbsc or autopsc or cbsct or ((autologous or auto-logous or auto or allogeneic or allo-geneic or homologous or homo-logous) adj hct)).ti,ab,kw	5068
4	or/1-3	277579
5	depression/ or exp depressive disorder/ or sadness/ or demoralization/ or mental health/ or happiness/ or hope/ or resilience, psychological/	24936

© 2024 Kulchycki M et al. *JAMA Network Open.*

6	(depress* or dysthymi* or melanchol* or affective or mood or distress* or sadness or sad or hopeless* or demorali* or mental health or psych* health or wellbeing or wellness or contented or contentment or happiness or hope* or resilien*).ti,ab,kw	155454
7	5 or 6	157208
8	exp exercise/ or exp exercise movement techniques/ or exp exercise therapy/ or dancing/ or exp physical fitness/ or exp sports/ or aquatic therapy/	42461
9	(exercis* or fitness or physiotherap* or physio therap* or kinesiotherap* or aerobics or athletic* or workout or gym? or gymnasi* or work* out or (activity adj4 prescri*)).ti,ab,kw	126288
10	(physical* adj4 (activ* or fit* or condition* or recondition* or program* or train*)).ti,ab,kw	48671
11	(activ* adj4 (living or play* or gaming or game* or lifestyle or behavio?r* or transport* or commut* or transit* or travel* or aerobic*)).ti,ab,kw	20674
12	(crossfit* or HIIT or HIIE or plyometric* or personal train* or bodybuild* or body build* or weightlift* or (weight* adj2 lift*)).ti,ab,kw	3350
13	((train* or condition* or program* or session*) adj5 (weight* or strength* or enduranc* or resistance or athletic* or cardiovascular or aerobic* or interval* or flexibility or balance or muscle* or muscular or circuit or isometric* or isotonic* or agility or power or propriocept* or coordination or gait)).ti,ab,kw	51938
14	(treadmill* or walk* or jog* or swim* or running or runner* or run or runs or marathon* or triathl* or dance or dancing or climb* or stair* or elliptical* or cycling or bicycl* or bike or bikes or calisthenic* or skateboard* or rowing or rower or canoe* or kayak* or paddl* or skate or skating or ski or skiing or hike or hiking or jump or jumping).ti,ab,kw	76765
15	(tai ji or tai chi or ai chi or yoga or pilates or stretch or stretches or stretching or flexibility or movement or qigong or qi gong or barre or tae bo or zumba or jazzercise).ti,ab,kw	46060
16	(exergam* or wii fit or zwift or peloton or kinect).ti,ab,kw	1061
17	(martial art* or boxing or kickboxing or karate or judo or jiu jitsu or jiujitsu or taekwondo or tae kwon do or fencing or wrestl* or aikido or fight* or grappl*).ti,ab,kw	2039
18	(sport* or golf* or aquatic* or gymnastic* or tennis or badminton or volleyball or ball or basketball or baseball or bowling or lacrosse or racquet* or ringette or softball or polo or cricket or hockey or football or soccer or rugby or handball or mountaineer*).ti,ab,kw	17091
19	or/8-18	247800
20	4 and 7 and 19	3341
21	limit 20 to (english language and yr="1980 -Current")	3119

Cinahl

© 2024 Kulchycki M et al. *JAMA Network Open.*

1	(MH neoplasms+) or (MH "cancer survivors") or (MH "psycho-oncology") or (MH "hematopoietic stem cell transplantation")	633417
2	TI(cancer* or neoplas* or tumor* or tumour* or malignan* or metasta* or oncogen* or oncolog* or psychooncolog* or sarcoma* or leukemi* or leukaemi* or lymphoma* or hodgkin* or nonhodgkin* or carcino* or melanoma* or thymoma* or myeloma* or blastoma* or hepatoblastoma* or mesenchymoma* or mesothelioma* or hepatoma* or adenocarcinoma* or glioma*) OR AB(cancer* or neoplas* or tumor* or tumour* or malignan* or metasta* or oncogen* or oncolog* or psychooncolog* or sarcoma* or leukemi* or leukaemi* or lymphoma* or hodgkin* or nonhodgkin* or carcino* or melanoma* or thymoma* or myeloma* or blastoma* or hepatoblastoma* or mesenchymoma* or mesothelioma* or hepatoma* or adenocarcinoma* or glioma*)	732757
3	TI(HSCT or ((h#ematopoietic or (h#emato N1 poietic)) N3 (transplant* or sct or bct or mobili?ation)) or pbsct or pbct or psct or ((peripheral or pbsc or pbscs) N3 transplant*) or autohct or autopbsct or autopbsc or autopsc or cbsct or ((autologous or "auto-logous" or auto or allogeneic or "allo-geneic" or homologous or "homo-logous") N1 hct)) OR AB(HSCT or ((h#ematopoietic or (h#emato N1 poietic)) N3 (transplant* or sct or bct or mobili?ation)) or pbsct or pbct or psct or ((peripheral or pbsc or pbscs) N3 transplant*) or autohct or autopbsct or autopbsc or autopsc or cbsct or ((autologous or "auto-logous" or auto or allogeneic or "allo-geneic" or homologous or "homo-logous") N1 hct))	7763
4	S1 or S2 or S3	875757
5	(MH depression+) or (MH sadness) or (MH demoralization) or (MH "mental health") or (MH happiness) or (MH hope) or (MH hopelessness) or (MH "psychological distress")	180871
6	TI(depress* or dysthymi* or melanchol* or affective or mood or distress* or sadness or sad or hopeless* or demorali* or "mental health" or (psych* N1 health) or wellbeing or wellness or contented or contentment or happiness or hope* or resilien*) OR AB(depress* or dysthymi* or melanchol* or affective or mood or distress* or sadness or sad or hopeless* or demorali* or "mental health" or (psych* N1 health) or wellbeing or wellness or contented or contentment or happiness or hope* or resilien*)	431571
7	S5 or S6	473887
8	(MH exercise+) or (MH "therapeutic exercise+") or (MH dancing+) or (MH "physical fitness+") or (MH sports+) or (MH "aquatic exercises") or (MH "gait training+")	226732
9	TI(exercis* or fitness or physiotherap* or (physio N1 therap*) or kinesiotherap* or aerobics or athletic* or workout or gym or gyms or gymnasi* or (work* N1 out) or (activity N4 prescri*)) OR AB(exercis* or fitness or physiotherap* or (physio N1 therap*) or kinesiotherap* or aerobics or athletic* or workout or gym or gyms or gymnasi* or (work* N1 out) or (activity N4 prescri*))	193329

© 2024 Kulchycki M et al. *JAMA Network Open.*

10	TI(physical* N4 (activ* or fit* or condition* or recondition* or program* or train*)) OR AB(physical* N4 (activ* or fit* or condition* or recondition* or program* or train*))	93921
11	TI(activ* N4 (living or play* or gaming or game* or lifestyle or behavio#r* or transport* or commut* or transit* or travel* or aerobic*)) OR AB(activ* N4 (living or play* or gaming or game* or lifestyle or behavio#r* or transport* or commut* or transit* or travel* or aerobic*))	49364
12	TI(crossfit* or HIIT or HIIE or plyometric* or (personal N1 train*) or bodybuild* or (body N1 build*) or weightlift* or (weight* N2 lift*)) OR AB(crossfit* or HIIT or HIIE or plyometric* or (personal N1 train*) or bodybuild* or (body N1 build*) or weightlift* or (weight* N2 lift*))	5025
13	TI((train* or condition* or program* or session*) N5 (weight* or strength* or enduranc* or resistance or athletic* or cardiovascular or aerobic* or interval* or flexibility or balance or muscle* or muscular or circuit or isometric* or isotonic* or agility or power or propriocept* or coordination or gait)) OR AB((train* or condition* or program* or session*) N5 (weight* or strength* or enduranc* or resistance or athletic* or cardiovascular or aerobic* or interval* or flexibility or balance or muscle* or muscular or circuit or isometric* or isotonic* or agility or power or propriocept* or coordination or gait))	60408
14	TI(treadmill* or walk* or jog* or swim* or running or runner* or run or runs or marathon* or triathl* or dance or dancing or climb* or stair* or elliptical* or cycling or bicycl* or bike or bikes or calisthenic* or skateboard* or rowing or rower or canoe* or kayak* or paddl* or skate or skating or ski or skiing or hike or hiking or jump or jumping) OR AB(treadmill* or walk* or jog* or swim* or running or runner* or run or runs or marathon* or triathl* or dance or dancing or climb* or stair* or elliptical* or cycling or bicycl* or bike or bikes or calisthenic* or skateboard* or rowing or rower or canoe* or kayak* or paddl* or skate or skating or ski or skiing or hike or hiking or jump or jumping)	135434
15	TI("tai ji" or "tai chi" or "ai chi" or yoga or pilates or stretch or stretches or stretching or flexibility or movement or qigong or "qi gong" or barre or "tae bo" or zumba or jazzercise) OR AB("tai ji" or "tai chi" or "ai chi" or yoga or pilates or stretch or stretches or stretching or flexibility or movement or qigong or "qi gong" or barre or "tae bo" or zumba or jazzercise)	107408
16	TI(exergam* or "wii fit" or zwift or peloton or kinect) OR AB(exergam* or "wii fit" or zwift or peloton or kinect)	1106
17	TI((martial N1 art*) or boxing or kickboxing or karate or judo or "jiu jitsu" or jiujitsu or taekwondo or "tae kwon do" or fencing or wrestl* or aikido or fight* or grappl*) OR AB((martial N1 art*) or boxing or kickboxing or karate or judo or "jiu jitsu" or jiujitsu or taekwondo or "tae kwon do" or fencing or wrestl* or aikido or fight* or grappl*)	20744

© 2024 Kulchycki M et al. *JAMA Network Open.*

18	TI(sport* or golf* or aquatic* or gymnastic* or tennis or badminton or volleyball or ball or basketball or baseball or bowling or lacrosse or racquet* or ringette or softball or polo or cricket or hockey or football or soccer or rugby or handball or mountaineer*) OR AB(sport* or golf* or aquatic* or gymnastic* or tennis or badminton or volleyball or ball or basketball or baseball or bowling or lacrosse or racquet* or ringette or softball or polo or cricket or hockey or football or soccer or rugby or handball or mountaineer*)	79711
19	S8 or S9 or S10 or S11 or S12 or S13 or S14 or S15 or S16 or S17 or S18	613585
20	(MH "randomized controlled trials+") or (MH "random assignment") or (MH "double-blind studies") or (MH "single-blind studies") or (MH "triple-blind studies")	201944
21	(ZT "clinical trial") or (ZT "randomized controlled trial")	241194
22	(clinical N1 trial*)	282744
23	(RCT or RCTs)	26927
24	((singl* or doubl* or treb* or tripl*) N1 (blind* or dumm* or mask*))	84817
25	(MH placebos)	13364
26	(placebo* or sham)	88259
27	AB(randomized or randomised or randomly)	316012
28	TI(trial)	164666
29	S20 or S21 or S22 or S23 or S24 or S25 or S26 or S27 or S28	617080
30	(MH vertebrates+) not (MH human)	212352
31	S29 not S30	591376
32	S4 and S7 and S19 and S31	1020
33	S32 Limiters - Published Date: 19800101-; English Language	1008
**Psychinfo**		
1	exp neoplasms/	57230
2	(cancer* or neoplas* or tumo?r* or malignan* or metasta* or oncogen* or oncolog* or psychooncolog* or sarcoma* or leuk?emi* or lymphoma* or hodgkin* or nonhodgkin* or carcino* or melanoma* or thymoma* or myeloma* or blastoma* or hepatoblastoma* or mesenchymoma* or mesothelioma* or hepatoma* or adenocarcinoma* or glioma*).ti,ab,id	95128
3	(HSCT or ((h?ematopoietic or h?emato-poietic) adj3 (transplant* or sct or bct or mobili#ation)) or pbsct or pbct or psct or ((peripheral or pbsc or pbscs) adj3 transplant*) or autohct or autopbsct or autopbsc or autopsc or cbsct or ((autologous or auto-logous or auto or allogeneic or allo-geneic or homologous or homo-logous) adj hct)).ti,ab,id	709
4	or/1-3	96531
5	"depression (emotion)"/ or contentment/ or distress/ or hapiness/ or hope/ or hopelessness/ or pessimism/ or sadness/ or exp major depression/ or demoralization/ or mental health/ or "resilience (psychological)"/	288996

© 2024 Kulchycki M et al. *JAMA Network Open.*

6	(depress* or dysthymi* or melanchol* or affective or mood or distress* or sadness or sad or hopeless* or demorali* or mental health or psych* health or wellbeing or wellness or contented or contentment or happiness or hope* or resilien*).ti,ab,id	798601
7	5 or 6	808004
8	exp physical activity/ or active living/ or movement therapy/ or dance/ or dance therapy/ or physical fitness/ or exp sports/ or athletic participation/ or athletic training/	90924
9	(exercis* or fitness or physiotherap* or physio therap* or kinesiotherap* or aerobics or athletic* or workout or gym? or gymnasi* or work* out or (activity adj4 prescri*)).ti,ab,id	106888
10	(physical* adj4 (activ* or fit* or condition* or recondition* or program* or train*)).ti,ab,id	57525
11	(activ* adj4 (living or play* or gaming or game* or lifestyle or behavio?r* or transport* or commut* or transit* or travel* or aerobic*)).ti,ab,id	56718
12	(crossfit* or HIIT or HIIE or plyometric* or personal train* or bodybuild* or body build* or weightlift* or (weight* adj2 lift*)).ti,ab,id	1956
13	((train* or condition* or program* or session*) adj5 (weight* or strength* or enduranc* or resistance or athletic* or cardiovascular or aerobic* or interval* or flexibility or balance or muscle* or muscular or circuit or isometric* or isotonic* or agility or power or propriocept* or coordination or gait)).ti,ab,id	32821
14	(treadmill* or walk* or jog* or swim* or running or runner* or run or runs or marathon* or triathl* or dance or dancing or climb* or stair* or elliptical* or cycling or bicycl* or bike or bikes or calisthenic* or skateboard* or rowing or rower or canoe* or kayak* or paddl* or skate or skating or ski or skiing or hike or hiking or jump or jumping).ti,ab,id	112096
15	(tai ji or tai chi or ai chi or yoga or pilates or stretch or stretches or stretching or flexibility or movement or qigong or qi gong or barre or tae bo or zumba or jazzercise).ti,ab,id	146396
16	(exergam* or wii fit or zwift or peloton or kinect).ti,ab,id	813
17	(martial art* or boxing or kickboxing or karate or judo or jiu jitsu or jiujitsu or taekwondo or tae kwon do or fencing or wrestl* or aikido or fight* or grappl*).ti,ab,id	22686
18	(sport* or golf* or aquatic* or gymnastic* or tennis or badminton or volleyball or ball or basketball or baseball or bowling or lacrosse or racquet* or ringette or softball or polo or cricket or hockey or football or soccer or rugby or handball or mountaineer*).ti,ab,id	54990
19	or/8-18	495157
20	exp randomized controlled trials/	1198
21	("0300").md	33511
22	(clinical adj trial$).tw,id	39520
23	(RCT or RCTs).tw,id	9914
24	((singl$ or doubl$ or treb$ or tripl$) adj (blind$3 or dumm* or mask$3)).tw,id	28049

© 2024 Kulchycki M et al. *JAMA Network Open.*

25	PLACEBO/	6232
26	(placebo$ or sham).tw,id	56395
27	(randomized or randomised or randomly).ab	160781
28	trial.ti	35090
29	or/20-28	236269
30	("20" not "10").po	377439
31	29 not 30	220516
32	4 and 7 and 19 and 31	414
33	limit 32 to (english language and yr="1980 -Current")	390

Scopus

1	TITLE-ABS-KEY(cancer* or neoplas* or tumo*r* or malignan* or metasta* or oncogen* or oncolog* or psychooncolog* or sarcoma* or leuk*emi* or lymphoma* or hodgkin* or nonhodgkin* or carcino* or melanoma* or thymoma* or myeloma* or blastoma* or hepatoblastoma* or mesenchymoma* or mesothelioma* or hepatoma* or adenocarcinoma* or glioma*)	6455147
2	TITLE-ABS-KEY(HSCT or ((h*ematopoietic or h*emato-poietic) W/3 (transplant* or sct or bct or mobili?ation)) or pbsct or pbct or psct or ((peripheral or pbsc or pbscs) W/3 transplant*) or autohct or autopbsct or autopbsc or autopsc or cbsct or ((autologous or auto-logous or auto or allogeneic or allo-geneic or homologous or homo-logous) W/1 hct))	89393
3	#1 or #2	6488041
4	TITLE-ABS-KEY(depress* or dysthymi* or melanchol* or affective or mood or distress* or sadness or sad or hopeless* or demorali* or "mental health" or (psych* W/1 health) or wellbeing or wellness or contented or contentment or happiness or hope* or resilien*)	2288241
5	TITLE-ABS-KEY(exercis* or fitness or physiotherap* or (physio W/1 therap*) or kinesiotherap* or aerobics or athletic* or workout or gym or gyms or gymnasi* or (work* W/1 out) or (activity W/4 prescri*))	1150296
6	TITLE-ABS-KEY(physical* W/4 (activ* or fit* or condition* or recondition* or program* or train*))	415073
7	TITLE-ABS-KEY(activ* W/4 (living or play* or gaming or game* or lifestyle or behavio*r* or transport* or commut* or transit* or travel* or aerobic*))	421786
8	TITLE-ABS-KEY(crossfit* or HIIT or HIIE or plyometric* or (personal W/1 train*) or bodybuild* or (body W/1 build*) or weightlift* or (weight* W/2 lift*))	26899
9	TITLE-ABS-KEY((train* or condition* or program* or session*) W/5 (weight* or strength* or enduranc* or resistance or athletic* or cardiovascular or aerobic* or interval* or flexibility or balance or muscle* or muscular or circuit or isometric* or isotonic* or agility or power or propriocept* or coordination or gait))	494382

© 2024 Kulchycki M et al. *JAMA Network Open.*

10	TITLE-ABS-KEY(treadmill* or walk* or jog* or swim* or running or runner* or run or runs or marathon* or triathl* or dance or dancing or climb* or stair* or elliptical* or cycling or bicycl* or bike or bikes or calisthenic* or skateboard* or rowing or rower or canoe* or kayak* or paddl* or skate or skating or ski or skiing or hike or hiking or jump or jumping)	2042429
11	TITLE-ABS-KEY("tai ji" or "tai chi" or "ai chi" or yoga or pilates or stretch or stretches or stretching or flexibility or movement or qigong or "qi gong" or barre or "tae bo" or zumba or jazzercise)	1944992
12	TITLE-ABS-KEY(exergam* or "wii fit" or zwift or peloton or kinect)	14406
13	TITLE-ABS-KEY((martial W/1 art*) or boxing or kickboxing or karate or judo or "jiu jitsu" or jiujitsu or taekwondo or "tae kwon do" or fencing or wrestl* or aikido or fight* or grappl*)	193326
14	TITLE-ABS-KEY(sport* or golf* or aquatic* or gymnastic* or tennis or badminton or volleyball or ball or basketball or baseball or bowling or lacrosse or racquet* or ringette or softball or polo or cricket or hockey or football or soccer or rugby or handball or mountaineer*)	747108
15	#5 or #6 or #7 or #8 or #9 or #10 or #11 or #12 or #13 or #14	6340866
16	TITLE-ABS-KEY(clinical W/1 trial*)	1654917
17	TITLE-ABS-KEY(RCT or RCTs)	73435
18	TITLE-ABS-KEY((singl* or doubl* or treb* or tripl*) W/1 (blind* or dumm* or mask*))	314512
19	TITLE-ABS-KEY(placebo* or sham)	532631
20	ABS(randomized or randomised or randomly)	1374881
21	TITLE(trial)	408099
22	#16 or #17 or #18 or #19 or #20 or #21	2945032
23	INDEXTERMS(animal*) AND NOT INDEXTERMS(human*)	5233875
24	#22 AND NOT #23	2721072
25	#3 AND #4 AND #15 AND #24	4540
26	#25 AND PUBYEAR AFT 1979 AND (LIMIT-TO (LANGUAGE , "English"))	4402

© 2024 Kulchycki M et al. *JAMA Network Open.*

eTable 2. Self-Reported Depression Assessment Scales Used in Included Trials

**Depression Scale**	**Number of Trials**
Center for Epidemiologic Studies Depression Scale (CES-D)	11
Hospital Anxiety and Depression Scale (HADS)	5
Beck Depression Inventory (BDI)	4
Profile of Mood States (POMS)	2
Profile of Mood States Short Form (POMS-SF)	1
Beck Depression Inventory II (BDI-II)	1
Linear Analog Self-Assessment (LASA)	1

© 2024 Kulchycki M et al. *JAMA Network Open.*

eFigure 1. Risk-of-Bias Domains for Each Included Study as per Cochrane Risk-of-Bias Tool 2

Green plus = low risk of bias. Yellow question mark = some concerns for bias. Red minus = high risk of bias. Overall bias is equal to the highest level of bias of the remaining five sections.

	Randomisation process	Intended intervention deviations	Missing outcome data	Measurement of the outcome	Selection of the reported result	Overall bias
Adams 2018	+	+	+	+	+	+
Burnham 2002	?	+	+	+	+	?
Cadmus 2009	+	+	+	+	+	+
Cantarero-Villaneuva 2013	+	−	+	+	+	−
Carter 2018	+	+	+	+	−	−
Cartmel 2021	+	+	+	+	+	+
Chang 2008	?	+	+	+	+	?
Chen 2015	+	+	−	+	+	−
Courneya 2003	?	+	+	+	+	?
Courneya 2007	+	+	+	+	+	+
Courneya 2009	+	+	+	+	+	+
Daley 2007	+	+	?	+	+	?
Dodd 2010	?	−	+	+	+	−
Eisenhut 2022	+	+	+	+	+	+
Ergun 2013	?	+	+	+	+	?
Gokal 2016	?	+	+	+	+	?
Ho 2016	+	+	?	+	+	?
Latka 2009	+	+	+	+	+	+
Monga 2007	?	−	?	+	+	−
Payne 2008	−	+	?	+	+	−
Pinto 2003	?	−	−	+	+	−
Piraux 2021	?	+	+	+	+	?
Piraux 2022	+	+	+	+	+	+
Rehman 2023	+	+	+	+	+	+
Saarto 2012	+	+	+	+	+	+

© 2024 Kulchycki M et al. *JAMA Network Open.*

eFigure 2. Summary of the Risk of Bias of Studies Included in the Systematic Review

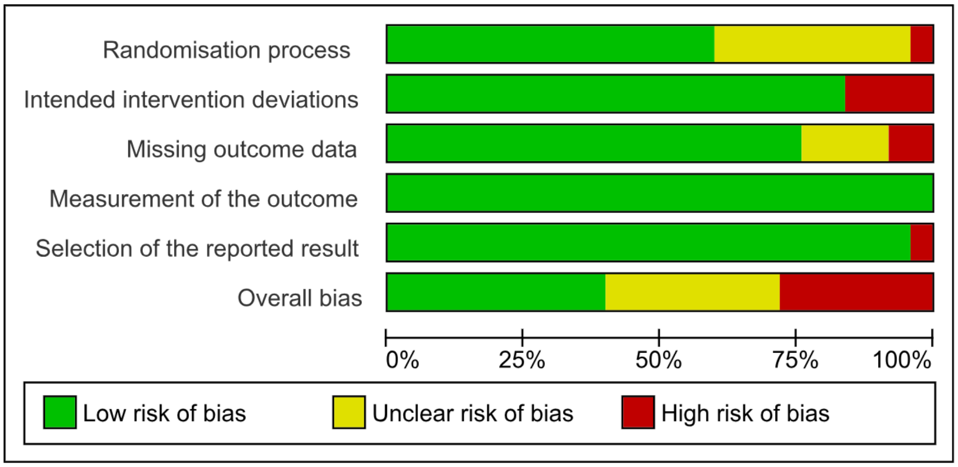

eFigure 3. Funnel Plot Assessing Publication Bias for Short-Term Reduction in the Severity of Depression

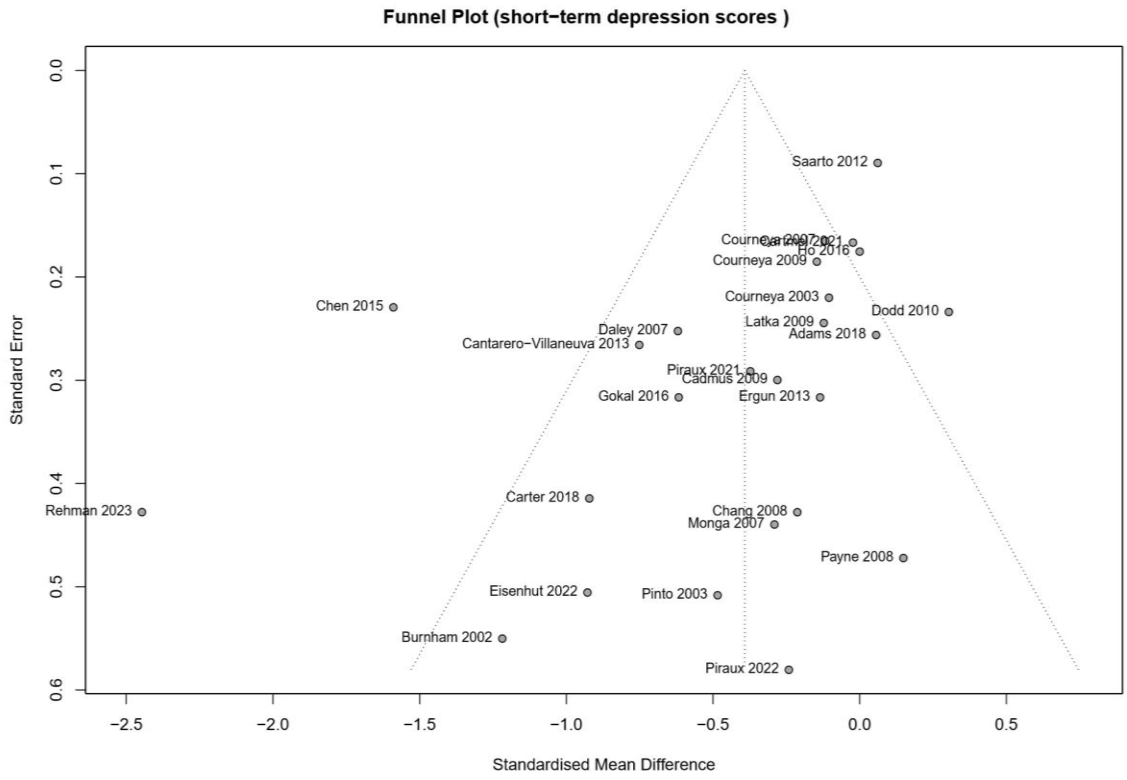

© 2024 Kulchycki M et al. *JAMA Network Open.*

eFigure 4. Forest Plot of Association of Aerobic Physical Activity (APA) With Short-Term Depression Including the Trials Using Validated Self-Reported Depression Scales

Diamonds refer to the aggregate standardized mean difference (SMD) and 95% confidence interval (CI) for that subgroup. Square size represents the relative weight of the study. Subtotals refer to overall effect in SMD with 95% CI.

	APA			Control			Std. Mean Difference	Weight
Study or Subgroup	Mean	SD	Total	Mean	SD	Total	IV, Random, (95% CI)	
**Validated Self-Reported Depression Scale**								
Rehman et al,[15] 2023	4.1	(1.7)	20	8.6	(1.9)	20	-2.45 (-3.28 to -1.61)	3.5%
Eisenhut et al,[16] 2022	7.8	(3.3)	10	10.7	(2.5)	8	-0.93 (-1.92 to 0.06)	2.9%
Piraux et al,[17] 2022	12	(3.2)	6	15	(15.9)	6	-0.24 (-1.38 to 0.90)	2.5%
Cartmel et al,[18] 2021	9.5	(8.6)	74	9.7	(8.9)	70	-0.02 (-0.35 to 0.30)	6.0%
Piraux et al,[19] 2021	10.5	(8.3)	24	13.9	(9.6)	24	-0.37 (-0.94 to 0.20)	4.8%
Adams et al,[20] 2018	4.2	(3.3)	35	4	(3.8)	27	0.06 (-0.45 to 0.56)	5.1%
Carter et al,[21] 2018	2.5	(2.2)	16	5.3	(3.8)	11	-0.92 (-1.73 to -0.11)	3.6%
Gokal et al,[34] 2016	4.4	(3.4)	21	6.2	(2.2)	21	-0.62 (-1.24 to 0.00)	4.5%
Ho et al,[35] 2016	5.5	(3.7)	66	5.5	(3.4)	64	0.00 (-0.34 to 0.34)	6.0%
Chen et al,[36] 2015	1.2	(3.1)	50	7.4	(4.5)	51	-1.59 (-2.04 to -1.14)	5.4%
Ergun et al,[38] 2013	3	(2.5)	20	3.3	(1.8)	20	-0.13 (-0.76 to 0.49)	4.5%
Saarto et al,[39] 2012	3.2	(3.2)	263	3	(3.3)	237	0.06 (-0.11 to 0.24)	6.6%
Dodd et al,[40] 2010	13	(9.6)	37	10.2	(8.6)	37	0.30 (-0.15 to 0.76)	5.4%
Cadmus et al,[41] 2009	7.9	(7.1)	22	10	(7.6)	23	-0.28 (-0.87 to 0.31)	4.7%
Couirneya et al,[42] 2009	5.4	(4.5)	57	6.1	(5)	60	-0.15 (-0.51 to 0.22)	5.9%
Latka et al,[43] 2009	9.6	(9.3)	34	10.8	(10.1)	33	-0.12 (-0.60 to 0.36)	5.3%
Payne et al,[45] 2008	12.7	(8.7)	9	11.4	(7.9)	9	0.15 (-0.78 to 1.07)	3.2%
Courneya et al,[46] 2007	9.7	(9.3)	74	10.8	(9.4)	73	-0.12 (-0.44 to 0.21)	6.1%
Daley et al,[47] 2007	6	(6.5)	33	10.3	(7.2)	33	-0.62 (-1.11 to -0.12)	5.2%
Monga et al,[48] 2007	2.8	(5.5)	11	4.2	(3.4)	10	-0.29 (-1.15 to 0.57)	3.4%
Courneya et al,[49] 2003	8.6	(8.7)	62	9.6	10.9	31	-0.10 (-0.54 to 0.33)	5.5%
**Total (95% CI)**			**944**			**868**	**-0.35 (-0.57 to -0.12)**	**100.0%**

Heterogeneity: $Tau^2 = 0.19$; $Chi^2 = 91.97$, df = 20 ($P < 0.00001$); $I^2 = 78\%$

Test for overall effect: Z = 3.04 (P = 0.002)

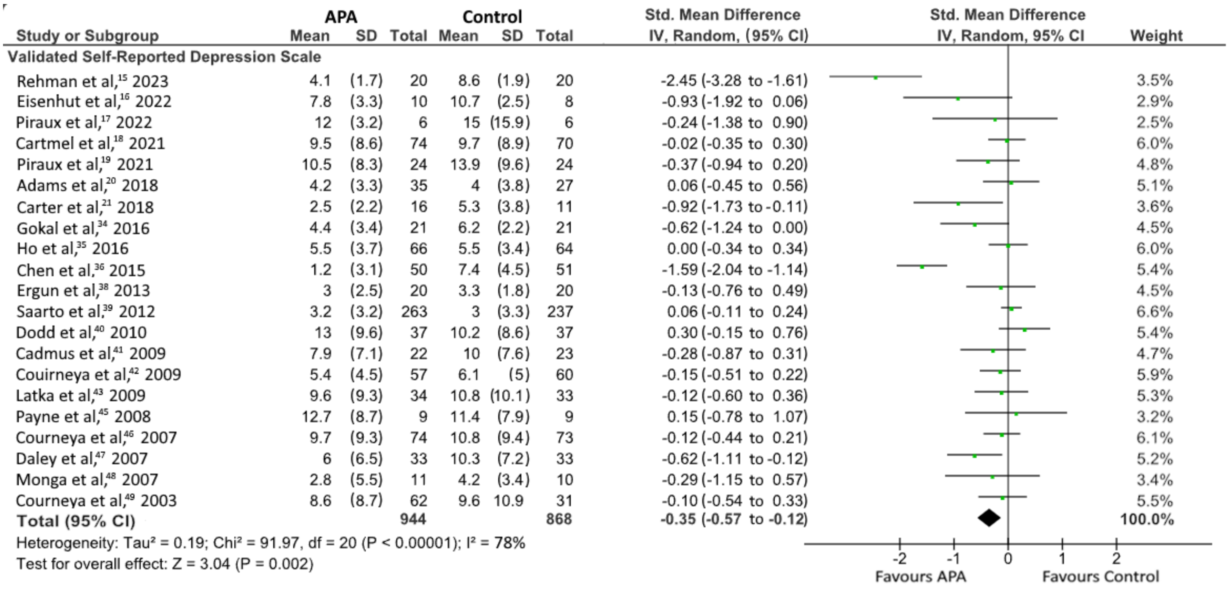

Validated self-reported depression scales included the Beck Depression Inventory (I & II) (BDI & BDI-II), the Center for Epidemiology Studies Depression Scale (CES-D), and the Hospital Anxiety and Depression Scale (HADS).

eFigure 5. Forest Plot of Association of Aerobic Physical Activity (APA) With Short-Term Depression Comparing the 3 Most Frequently Validated Self-Reported Depression Scales Used by the Trials

Diamonds refer to the aggregate standardized mean difference (SMD) and 95% confidence interval (CI) for that subgroup. Square size represents the relative weight of the study. Subtotals refer to overall effect in SMD with 95% CI.

	Aerobic PA			Control			Std. Mean Difference		Weight
Study or Subgroup	Mean	(SD)	Total	Mean	(SD)	Total	IV, Random, (95% CI)	Year	
**Center For Epidemiologic Studies Depression Scale (CES-D)**									
Piraux 2022	12	(3.2)	6	15	(15.9)	6	-0.24 (-1.38 to 0.90)	2022	2.6%
Piraux 2021	10.5	(8.3)	24	13.9	(9.6)	24	-0.37 (-0.94 to 0.20)	2021	5.0%
Cartmel 2021	9.5	(8.6)	74	9.7	(8.9)	70	-0.02 (-0.35 to 0.30)	2021	6.4%
Adams 2018	4.2	(3.3)	35	4	(3.8)	27	0.06 (-0.45 to 0.56)	2018	5.4%
Dodd 2010	13	(9.6)	37	10.2	(8.6)	37	0.30 (-0.15 to 0.76)	2010	5.7%
Cadmus 2009	7.9	(7.1)	22	10	(7.6)	23	-0.28 (-0.87 to 0.31)	2009	4.9%
Courneya 2009	5.4	(4.5)	57	6.1	(5)	60	-0.15 (-0.51 to 0.22)	2009	6.2%
Latka 2009	9.6	(9.3)	34	10.8	(10.1)	33	-0.12 (-0.60 to 0.36)	2009	5.5%
Payne 2008	12.7	(8.7)	9	11.4	(7.9)	9	0.15 (-0.78 to 1.07)	2008	3.4%
Courneya 2007	9.7	(9.3)	74	10.8	(9.4)	73	-0.12 (-0.44 to 0.21)	2007	6.4%
Courneya 2003	8.6	(8.7)	62	9.6	(10.9)	31	-0.10 (-0.54 to 0.33)	2003	5.8%
**Subtotal (95% CI)**			**434**			**393**	**-0.07 (-0.21 to 0.07)**		**57.3%**
Heterogeneity: Tau² = 0.00; Chi² = 5.07, df = 10 (P = 0.89); I² = 0%									
Test for overall effect: Z = 1.03 (P = 0.30)									
**Hospital Anxiety and Depression Scale (HADS)**									
Rehman 2023	4.1	(1.7)	20	8.6	(1.9)	20	-2.45 (-3.28 to -1.61)	2023	3.7%
Carter 2018	2.5	(2.2)	16	5.3	(3.8)	11	-0.92 (-1.73 to -0.11)	2018	3.8%
Gokal 2016	4.4	(3.4)	21	6.2	(2.2)	21	-0.62 (-1.24 to 0.00)	2016	4.8%
Ho 2016	5.5	(3.7)	66	5.5	(3.4)	64	0.00 (-0.34 to 0.34)	2016	6.3%
Chen 2015	1.2	(3.1)	50	7.4	(4.5)	51	-1.59 (-2.04 to -1.14)	2015	5.7%
**Subtotal (95% CI)**			**173**			**167**	**-1.08 (-1.94 to -0.22)**		**24.3%**
Heterogeneity: Tau² = 0.86; Chi² = 47.74, df = 4 (P < 0.00001); I² = 92%									
Test for overall effect: Z = 2.47 (P = 0.01)									
**Beck Depression Inventory I & II (BDI I & II)**									
Eisenhut 2022	7.8	(3.3)	10	10.7	(2.5)	8	-0.93 (-1.92 to 0.06)	2022	2.9%
Ergun 2013	3	(2.5)	20	3.3	(1.8)	20	-0.13 (-0.76 to 0.49)	2013	4.5%
Saarto 2012	3.2	(3.2)	263	3	(3.3)	237	0.06 (-0.11 to 0.24)	2012	6.6%
Daley 2007	6	(6.5)	33	10.3	(7.2)	33	-0.62 (-1.11 to -0.12)	2007	5.2%
Monga 2007	2.8	(5.5)	11	4.2	(3.4)	10	-0.29 (-1.15 to 0.57)	2007	3.4%
**Subtotal (95% CI)**			**337**			**308**	**-0.28 (-0.66 to 0.10)**		**22.7%**
Heterogeneity: Tau² = 0.10; Chi² = 10.03, df = 4 (P = 0.05); I² = 60%									
Test for overall effect: Z = 1.45 (P = 0.15)									
**Total (95% CI)**			**911**			**835**	**-0.33 (-0.56 to -0.10)**		**100.0%**
Heterogeneity: Tau² = 0.19; Chi² = 88.83, df = 19 (P < 0.00001); I² = 79%									
Test for overall effect: Z = 2.82 (P = 0.005)									
Test for subgroup differences: Chi² = 5.20, df = 2 (P = 0.07), I² = 61.5%									

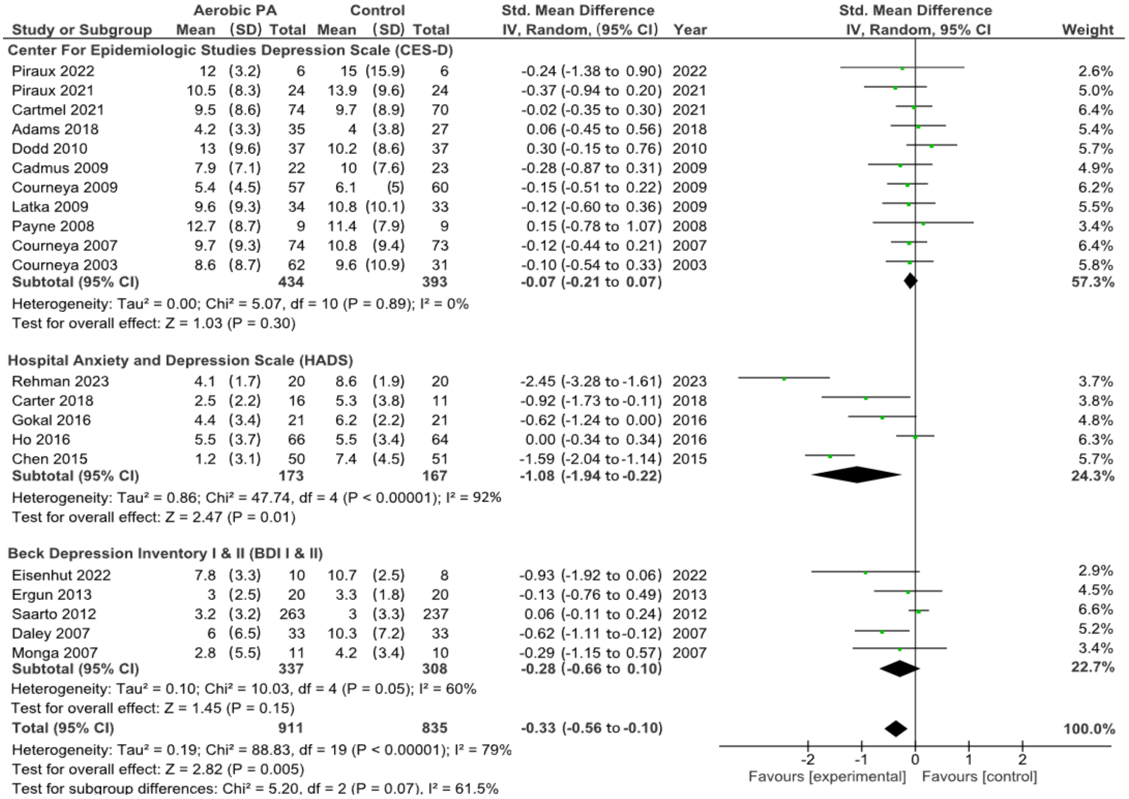

© 2024 Kulchycki M et al. *JAMA Network Open.*
