# Supplementary material for: Aerobic Physical Activity and Depression Among Patients With Cancer: A Systematic Review and Meta-Analysis
Source: JAMA Netw Open. 2024 Oct 8;7(10):e2437964. doi: 10.1001/jamanetworkopen.2024.37964 (PMC11581595; doi:10.1001/jamanetworkopen.2024.37964)
Supplement: Supplement 2. — Data Sharing Statement [file jamanetwopen-e2437964-s002.pdf]

Data Sharing Statement

Kulchycki. Aerobic Physical Activity and Depression Among Patients With Cancer. *JAMA Netw Open*. Published October 08, 2024. doi:10.1001/jamanetworkopen.2024.37964

Data
Data available: No
